# Relationships between birth or calving seasons and first-lactation performance of Holstein cows in the Midwestern United States

**DOI:** 10.3168/jdsc.2025-0764

**Published:** 2025-05-22

**Authors:** K.N. Brost, J.K. Drackley

**Affiliations:** Department of Animal Sciences University of Illinois, Urbana, IL 61801

## Abstract

The purpose of this study was to establish the effects of birth season on performance of first-lactation Holstein cows. Further analysis was conducted to determine if the impact of calving season was a more appropriate indicator. This study analyzed data from 2009 to 2022 and included 524 primiparous Holstein cows from the University of Illinois Urbana-Champaign Dairy Research Unit (Urbana, IL). Cows were managed per farm protocols. Seasons were assigned by birth and calving months: winter (December, January, February), spring (March, April, May), summer (June, July, August), and fall (September, October, November). The main comparison was winter versus summer seasons of birth and calving, but summer versus non-summer and winter versus non-winter were also used. Data were analyzed using the MIXED, UNIVARIATE, and MEANS procedures in SAS v. 9.4. Based on the first-lactation data, average test day milk yield (kg) for d 5 to 35 was less for summer-born cows in comparison to winter and non-summer-born cows. Average first test day fat and protein percentages were less for summer-born cows in contrast to winter. The length of pregnancy was less for summer-born cows compared with non-summer. The age at first calving (AFC) was lower for summer-born cows in comparison to winter and non-summer, whereas winter was greater than non-winter. No differences were observed for the number of times bred, number of lactations, or 305-d milk yield. Summer-calving cows tended to have less first test day milk yield (kg) compared with non-summer. First test day fat and protein percentages were lower for summer in comparison to winter and non-summer, whereas winter was greater than non-winter. Days pregnant were fewer for summer-calving cows relative to winter. Summer-calving cows stayed in the herd for fewer lactations than winter and non-summer, however winter-calving cows stayed for a greater number of lactations. Cows that calved in winter and summer tended to have lower AFC relative to non-winter and non-summer-calving cows, respectively. No differences were observed in times bred or 305-d milk yield. Overall, an association between birth and calving seasons was observed, suggesting their influence on first-lactation performance. Birth season data were more closely related to first test day milk yield and AFC, whereas calving season had a greater effect on first test day milk components and productive life, implying greater focus should be placed on calf husbandry and first-lactation cows calving during the warmest season in the Midwestern United States.

## Highlights

• First test day milk yield and age at first calving were lowest for summer-born cows.
• First test day fat and protein percentages were lowest for summer-born and calving cows.
• Production life was shortest for summer-calving cows.
• Summer-born and calving cows had or tended to have shorter pregnancy lengths.
• There are opportunities to advance management of summer-born and calving cows.

## Graphical Abstract

**Figure:**
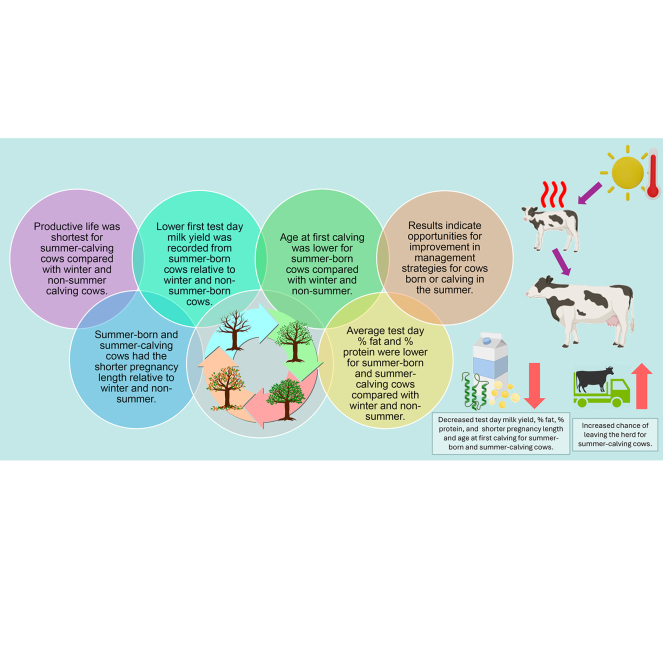
**Summary:** The purpose of this analysis was to establish the effects of birth season on the performance of first-lactation Holstein cows. Further analysis was conducted to determine the impact of calving season. This study analyzed data from 2009 to 2022 and included 524 first-parity Holstein cows from the University of Illinois Dairy Research Unit. An association between birth and calving seasons in relation to first-lactation cow performance was observed. A pattern of reduced milk production and reproductive performance was noted for summer-born and summer-calving cows. These data indicate opportunities for improvement in feeding and management strategies for cows born or calving during the warmer summer season.

Birth and calving seasons have been observed to influence the first-lactation production of dairy cows. According to Barash et al. (1996), birth month had a greater effect on first-parity cows and became less important in later parities compared with calving month. Calving season was found to have a greater effect on first-lactation performance than birth season according to Van Eetvelde et al. (2020). Chester-Jones et al. (2017) observed that calf BW and ADG at 6 and 8 wk had positive effects on 305-d first-lactation milk yield, fat percentage, and protein percentage, whereas starter DMI at 8 wk enhanced first-lactation performance. These data may indicate improved first-lactation performance when seasonal differences in BW, ADG, and DMI are present. Previous findings on the impacts of birth or calving season on performance were mixed. Some research reported that summer-born calves in temperate and continental climates had increased 305-d milk, fat, and protein yields, particularly in herds where calves were fed consistent amounts of milk throughout the year (Soberon et al., 2012; Van Eetvelde et al., 2017). Temperate climate refers to regions with 4 distinct seasons and moderate temperatures, whereas a continental climate is characterized by 4 distinct seasons with substantial temperature variations from cold winters to hot summers. Other research disagrees, stating that winter-born calves had increased DMI and ADG, and that milk fat, protein, and total solids percentages were the lowest during the summer months and the highest during the winter (Ozrenk and Inci, 2008; Chester-Jones et al., 2017; Van Eetvelde et al., 2020). Data from continental and semi-arid regions noted reduced milk production and reproductive performance for summer-calving cows (Sargeant et al.,1998; Ray et al., 1992). Semiarid climates host drier conditions with hot summers and cool winters. Although differences could be expected in varying climates, further discrepancies could be due to management and feeding protocols. Strategies to help mitigate the impacts of heat stress could include providing shade, increasing ventilation, using misters, changing feeding times, and appropriately using minerals in diets (West, 2003; Ozrenk and Inci, 2008). Inconsistent results indicate a need for additional regional, climate-specific research to better adapt husbandry practices to ensure ideal calf growth, optimal milk production, and reproductive performance of dairy herds. Our aim was to determine the effects of birth and calving season on performance of first-lactation Holstein cows.

This study included 524 first parity Holstein dairy cows from the University of Illinois Urbana-Champaign Dairy Research Unit (Urbana, IL). Data from PC Dart (Dairy Records Management Systems, Raleigh, NC) were analyzed from 2009 to 2022. Daily care was provided by farm staff. Cows were assigned a season based on month of birth or calving: winter (n = 156; December, January, February), spring (n = 112; March, April, May), summer (n = 119; June, July, August), and fall (n = 137; September, October, November). Reproductive and herd life assessments were based on the number of times bred, length of pregnancy, age at first calving (**AFC**), and the productive life length of the cows. Production data from milk yield (kg), fat and protein percentages of test day milk from d 5 to 35, and 305-d milk yield were evaluated for seasonal variations. First test day milk yield, fat percentage, and protein percentage were used for their relationship to future milk yield, disease, pregnancy risk, and culling (Heuer et al., 1999; Cook and Green, 2016). Days 5 to 35 of first test day data were used in this analysis due to the lack of data on cows before d 5 from colostrum collection and disposal of transition milk. The end point of d 35 was used to include cows during the fresh period and was an average of time periods used in previous research (Heuer et al., 1999; Cook and Green, 2016).

This analysis was a quasi-experimental design. Cow was the experimental unit (n = 524), and year was a random effect. Confounding of birth and calving season was corrected for by using one variable as a covariate when the other was analyzed, adjusting the model for the effect of the other factor. The main comparisons were winter versus summer, summer versus non-summer, and winter versus non-winter, where non-summer and non-winter calves include all seasons not stated in the name. Data from cows that produced twins or left the herd before completing a lactation were included in the analysis. Data were analyzed using the MIXED, UNIVARIATE, and MEANS procedures in SAS v. 9.4 (SAS Institute Inc., Cary, NC). Significance was declared at *P* < 0.05 and trends were discussed when 0.05 < *P* ≤ 0.10.

Although birth and calving seasons are often similar, particularly for first-lactation cows, there are distinct differences. Of the 524 cows used in the current analysis, 259 shared the same birth and calving season, 263 cows had different birth and calving seasons, and 2 cows did not have a calving season recorded. Barash et al. (1996) stated the importance of birth month on milk production, fat, and protein specifically to first-lactation cows, whereas calving month had a greater effect of later parity cows. The current study observed differences in both birth and calving season. Birth season data are shown in Table 1. On average, first test day milk yield was lower for summer-born cows compared with winter (*P* = 0.004) and to non-summer (*P* = 0.002) and tended to be greater (*P* = 0.10) for winter-born cows relative to non-winter. According to Heuer et al. (1999), there are relationships between first test day milk yield and noninfectious diseases, 100-d milk yield, and culling, with milk yield being greater for cows with hypocalcemia and lower for cows with displaced abomasum, retained placenta, and metritis. The authors explained that the increased milk yield of cows with hypocalcemia may be due to a greater risk of milk fever with high-producing cows. They also stated that diagnostics that used first test day data may significantly improve, and that first test day milk yield is a better indicator of those traits compared with BCS or loss of body condition between the first and second scoring after calving. First-lactation milk yield has also been associated with preweaning growth rates (Soberon et al., 2012). Cook and Green (2016) observed average milk yield during the fourth week of lactation could be used as an early indicator of production and a positive association was identified between that and the potential for pregnancy at both 100 and 150 DIM. Average first test day fat percentage was lower for summer-born cows relative to winter (*P* = 0.005) and tended to be lower for non-summer (*P* = 0.08), whereas it was greater for winter-born cows (*P* = 0.04) compared with non-winter. The average first test day protein percentage was lower for summer-born cows compared with winter (*P* = 0.04), whereas it tended to be greater for winter-born cows (*P* = 0.09) than non-winter, and summer tended to be lower in comparison to non-summer (*P* = 0.10). Increased protein percentage during early lactation is associated with the predicted probability of pregnancy at 100 DIM and 150 DIM, whereas increased fat percentage was negatively associated with probability of pregnancy at 100 DIM but not at 150 DIM. Although early-lactation fat percentage may be more limited in predicting risk of pregnancy, it can be suggestive of energy balance issues (Cook and Green, 2016). Our data agree with previous results from Ozrenk and Inci (2008), who stated that fat and protein percentages were greatest during the winter and the lowest during the summer. However, this diverges from research by Chester-Jones et al. (2017), who observed greater percentages of fat and protein for summer-born heifers.

**Table 1:** Birth season means for 305-d milk yield, test day milk yield (d 5–35), milk fat percentage and protein percentage (d 5–35), number of days pregnant and lactations, times bred, and AFC for first-lactation cows, with contrasts focusing on winter and summer seasons

Variable	Season^1^				SE	Contrast^2^ (*P*-value)		
	Winter	Spring	Summer	Fall		Winter vs. summer	Winter vs. non-winter	Summer vs. non-summer
305-d milk yield, kg	10,315	10,406	10,351	10,504	76.6	0.88	0.56	0.77
Test day milk yield d 5–35, kg	30.64	31.24	27.29	28.97	0.38	0.004	0.10	0.002
Milk fat %	4.28	3.90	3.94	4.19	0.04	0.005	0.004	0.08
Milk protein %	3.16	3.05	3.05	3.17	0.05	0.04	0.09	0.10
Pregnancy length, d	276.4	276.6	274.8	276.2	0.31	0.10	0.49	0.05
Number of lactations	2.77	2.71	2.63	2.85	0.06	0.41	0.75	0.30
Times bred	1.73	1.64	1.79	1.74	0.04	0.64	0.97	0.44
AFC, mo (± SD)	24.34 ± 1.54	24.37 ± 2.31	23.71 ± 1.75	23.85 ± 1.74	0.08	0.004	0.03	0.01

1 Season: winter = December, January, February (n = 156); spring = March, April, May (n = 112); summer = June, July, August (n = 119); fall = September, October, November (n = 137).

2 Contrasts: non-winter = all months not included in winter; non-summer = all months not included in summer.

Age at first calving (mo) was greater for winter-born cows in comparison to summer-born cows (*P* = 0.004) and non-winter (*P* = 0.03) and was lower for summer-born (*P* = 0.01) relative to non-summer-born cows. Average AFC were 24.34 ± 1.54 mo for winter, 24.37 ± 2.31 mo for spring, 23.71 ± 1.75 mo for summer, and 23.85 ± 1.74 mo for fall, which align with previous research suggesting the optimum AFC is 22 to 25 mo (Pirlo et al., 2000; Ettema and Santos, 2004; Eastham et al., 2018). The length of pregnancy was shorter for summer-born cows (*P* = 0.05) compared with non-summer and tended to be shorter for summer (*P* = 0.10) in relation to winter. No differences were observed for the number of lactations among the seasonal comparisons. This disagrees with previous work by Toledo et al. (2024), who observed an increase in productive life for cows born in cool months in California and an increase in cows sold that were born during hot months in Florida. The authors emphasized that an increase in productive life can decrease costs associated with heifer rearing and reduce environmental impacts, further highlighting the importance of extending productive life. The number of times bred did not differ in the current study, whereas research by Toledo et al. (2024) reported more cows sold that were born during hot months due to breeding issues. No differences were noted in 305-d milk yield. The 305-d milk yield data from this study deviate from earlier research that observed lower first-lactation milk yields for cows born in either summer or winter (Chester-Jones et al., 2017; Van Eetvelde et al., 2017, 2020). Although greater early-life BW and ADG are thought to increase 305-d milk yield and milk components, the opposite was observed by Chester-Jones et al. (2017), with greater BW and ADG in fall and winter, but greater milk yield and components noted during summer. Prenatal temperature-humidity index (**THI**) affects the birth weight of calves; birth weights of calves with low THI values near the end of gestation were higher than the calves with high THI values for the same period (Yaylak et al., 2015). The differences could be explained by management practices such as nutrition and photoperiod. A further explanation for the possible decrease in milk yield for summer-born heifers is heat stress. The intrauterine environment may be altered due to late-gestation heat stress, resulting in different metabolic and mammary phenotypes in adulthood (Laporta et al., 2020). Skibiel et al. (2018) reported smaller mammary alveoli consisting of fewer secretory cells for in utero heat-stressed heifers relative to those born to cooled dams. This suggests that intrauterine heat stress exposure could modify the course of development and cause inferior lifetime performance for calves born to heat-stressed dams (Laporta et al., 2020). Although not part of the current analyses, intrauterine and prenatal heat stress may have delayed mammary development, explaining the differences in first test day and 305-d milk yields.

Although birth month has been observed to influence cows' first-lactation production, the current analysis noted that calving season had a substantial impact as well. This aligns with a study by Van Eetvelde et al. (2020), who reported calving season to have a greater effect on first-lactation performance compared with birth season, though in the current analyses birth and calving seasons were not directly compared. Calving season results are in Table 2. Summer-calving cows tended to have a lower average first test day milk yield compared with non-summer (*P* = 0.07). Average first test day fat percentage was lower for summer relative to winter (*P* = 0.006) and summer relative to non-summer (*P* = 0.05), whereas winter-calving cows had a greater average fat percentage (*P* = 0.009) compared with non-winter. Similarities continued for average first test day protein percentage, with summer-calving cows producing less compared with winter (*P* = 0.001) and non-summer (*P* = 0.007), and winter being greater compared with non-winter (*P* = 0.008). These results are concurrent with previous research reporting lower milk yield in first-lactation heifers that calved in the summer (Maciuc, 2009; Mohd Nor et al., 2013). Decreases in summer milk yield could be caused by cows attempting to lower their metabolic heat production by decreasing DMI during warmer weather. This reduction in intake during the early stage of lactation could potentially depress summer milk yields (Maciuc, 2009).

**Table 2:** Calving season means for 305-d milk (kg), test day milk yield (d 5–35), milk fat percentage and protein percentage (d 5–35), number of days pregnant and lactations, times bred, and AFC for first-lactation cows, with contrasts focusing on winter and summer seasons

Variable	Season^1^				SE	Contrast^2^ (*P*-value)		
	Winter	Spring	Summer	Fall		Winter vs. summer	Winter vs. non-winter	Summer vs. non-summer
305-d milk yield, kg	10,292	10,461	10,309	10,521	76.5	0.94	0.47	0.56
Test day milk yield d 5–35, kg	29.89	31.02	28.31	29.41	0.38	0.19	0.74	0.07
Milk fat, %	4.27	3.88	3.92	4.24	0.04	0.006	0.009	0.05
Milk protein, %	3.19	2.99	3.01	3.22	0.02	0.001	0.008	0.007
Pregnancy length, d	276.9	275.5	274.4	277.0	0.31	0.01	0.09	0.009
Number of lactations	2.94	2.65	2.51	2.81	0.06	0.02	0.04	0.05
Times bred	1.77	1.77	1.65	1.71	0.04	0.38	0.58	0.36
AFC, mo (± SD)	23.89 ± 1.61	24.22 ± 2.13	23.87 ± 1.73	24.43 ± 1.87	0.08	0.96	0.10	0.10

1 Season: winter = December, January, February (n = 155); spring = March, April, May (n = 104); summer = June, July, August (n = 129); fall = September, October, November (n = 134).

2 Contrasts: non-winter = all months not included in winter; non-summer = all months not included in summer.

Age at first calving tended to be lower (*P* = 0.10) for both winter-calving in comparison to non-winter cows and summer-calving compared with non-summer cows. Average AFC were 23.89 ± 1.61 mo for winter, 24.22 ± 2.13 mo for spring, 23.87 ± 1.73 mo for summer, and 24.43 ± 1.87 mo for fall. Days pregnant were lower for summer-calving cows compared with winter (*P* = 0.01) and non-summer (*P* = 0.009) and tended to be greater for winter relative to non-winter (*P* = 0.09). This agrees with previous research by Vieira-Neto et al. (2017), who reported cows calving during the hot season had a pregnancy length 1.5 d shorter than cows calving during the cool season (hot = 274.7 vs. cool = 276.2 d). The authors also observed that cows with shorter pregnancy length had increased incidence of reproductive disorders following calving compared with average pregnancy length, resulting in greater morbidity in the first 90 DIM and culling by 300 DIM, with an overall faster rate of removal from the herd. Summer-calving cows stayed in the herd fewer average lactations than winter (*P* = 0.02) and non-summer (*P* = 0.05), whereas greater average lactations were recorded for winter relative to non-winter (*P* = 0.04). Shorter productive life for summer-calving cows is supported by the aforementioned reproductive disorders and overall faster removal from the herd (Vieira-Neto et al., 2017). As previously stated, cows may voluntarily decrease DMI during warmer temperatures to reduce metabolic temperature, leading to depressed milk production, especially in early lactation (Maciuc, 2009). Reduced DMI in fresh cows may lead to metabolic disorders such as ketosis, subacute ruminal acidosis, and hypocalcemia. Although these conditions can be managed, affected animals are still at risk due to the overall impact of decreased DMI on the immune system and potential increased susceptibility to other diseases. Health factors such as these can increase voluntary and involuntary culling rates of first-lactation cows, but health analysis was not part of this study. No differences were identified in times bred or for 305-d milk yield.

In all, an association between birth or calving seasons in relation to first-lactation cow performance was observed. A pattern of reduced first test day milk production and components, pregnancy length, and AFC were revealed for summer-born cows, whereas data from summer-calving cows displayed reductions in first test day milk production and components, pregnancy length, productive life, and AFC. These data indicate opportunities for improvement in feeding and management strategies for cows born or calving during the warmer summer season. Heat mitigation for close-up and fresh cows, as well as calves, and improved feeding techniques for calves and heifers during periods of heat stress, could improve the overall performance and productive life of dairy herds in the Midwestern United States.
